# Clinical application guidelines for blood glucose monitoring in China (2022 edition)

**DOI:** 10.1002/dmrr.3581

**Published:** 2022-10-25

**Authors:** Yuqian Bao, Dalong Zhu, Chinese Diabetes Society

**Affiliations:** ^1^ Department of Endocrinology and Metabolism Shanghai Sixth People's Hospital Affiliated to Shanghai Jiao Tong University School of Medicine Shanghai China; ^2^ Department of Endocrinology Drum Tower Hospital Affiliated to Nanjing University Medical School Nanjing China

**Keywords:** diabetes, glucose monitoring, guideline

## Abstract

Glucose monitoring is an important component of diabetes management. The Chinese Diabetes Society (CDS) has been producing evidence‐based guidelines on the optimal use of glucose monitoring since 2011. In recent years, new technologies in glucose monitoring and more clinical evidence, especially those derived from Chinese populations, have emerged. In this context, the CDS organised experts to revise the *Clinical application guidelines for blood glucose monitoring in China* in 2021. In this guideline, we focus on four methods of glucose monitoring that are commonly used in clinical practice, including capillary glucose monitoring, glycated haemoglobin A1c, glycated albumin, and continuous glucose monitoring. We describe the definitions and technical characteristics of these methods, the factor that may interfere with the measurement, the advantages and caveats in clinical practice, the interpretation of glucose metrics, and the relevant supporting evidence. The recommendations for the use of these methods are also provided. The various methods of glucose monitoring have their strengths and limitations and cannot be replaced by one another. We hope that these guidelines could aid in the optimal application of common methods of glucose monitoring in clinical practice for better diabetes care.

## 1 INTRODUCTION

Glucose monitoring, as an important part of diabetes management, serves to evaluate the glucose status and the effectiveness of glucose lowering therapies. Common methods of glucose monitoring that are used in clinical practice include capillary glucose monitoring (which includes self‐monitoring of blood glucose [SMBG] and the point‐of‐care testing [POCT] in hospital), glycated haemoglobin A1c (HbA1c), glycated albumin (GA), and continuous glucose monitoring (CGM). In 2011, the Chinese Diabetes Society (CDS) issued the *Clinical application guideline for blood glucose monitoring in China (2011 Edition)* (hereinafter referred to as the *guideline*), and further revision was made in 2015. In recent years, glucose monitoring technology is evolving in a more convenient, accurate, and minimally invasive or even noninvasive direction. In order to further standardise the use of various tools based on the recent advancements in technologies and evidence, the *guideline* is revised and updated again.

The *guideline* lists the recommendations at the beginning of each chapter and marks the level of evidence (Table 1). Evidence level A is the evidence based on multiple randomised controlled trials or meta‐analysis; evidence level B is the evidence based on a single randomised controlled trial or multiple non‐randomised controlled trials; and evidence level C is the evidence only based on expert consensus and/or small‐scale studies, retrospective studies, and results from registration studies.

**TABLE 1:** Level of evidence

Level A	Evidence from multiple randomised controlled trials or meta‐analysis of randomised controlled trials
Level B	Evidence from a single randomised controlled trial or multiple non‐randomised controlled trials
Level C	Expert consensus and/or evidence from small‐scale studies, retrospective studies, or registration studies

## 2 CAPILLARY GLUCOSE MONITORING

*Recommendations*

1. SMBG is an integral part of diabetes management and education. It is recommended that all patients with diabetes should perform SMBG (A).
2. Individualised protocols of capillary glucose monitoring should be made according to the practical needs of patients with diabetes (B).

Capillary glucose monitoring includes SMBG and POCT (in medical institutions), which is the most basic and effective way for daily management. It can reflect real‐time glucose levels. It can also be used to evaluate the effects of life events (such as diet, exercise, mood, stress etc.), diseases, and medications on glucose levels, which can help improve the effectiveness and safety of treatment and improve patients' quality of life. However, capillary glucose values cannot be used for the diagnosis of diabetes.

### 2.1 SMBG

As part of self‐management, SMBG can help patients better understand their glucose status, provide a way to actively participate in diabetes management, and adjust lifestyle and medications accordingly. SMBG is an integral component of comprehensive diabetes management and education. The guidelines issued by the International Diabetes Federation,^1^ the American Diabetes Association,^2^ and the National Institute for Health and Care Excellence in the UK^3^ all recommend that patients with diabetes should perform SMBG as needed, especially those receiving insulin therapy. The use of SMBG can improve metabolic control and may reduce the risk of diabetes‐related outcomes.

### 2.2 POCT in hospitals

In medical institutions (hereinafter referred to as ‘in hospitals’), glucose monitoring can be done through the central laboratory using an automatic biochemical analyser to measure venous plasma or serum glucose levels. But in more cases, glucose monitoring is completed through POCT. In the same site, clinic, or inpatient area, in principle, the same type of glucose monitoring equipment should be used to avoid deviations in test results caused by performance differences between different glucose testing systems. The glucose metres used by medical institutions should comply with the accuracy and precision requirements as follows. Currently, intelligent systems are increasingly adopted for glucose monitoring in hospitals. Such systems can identify the patients' information and automatically transmit glycaemic results, thereby avoiding manual errors while improving efficiency. Moreover, all glycaemic results and records of quality control can be tracked.

### 2.3 Influencing factors of capillary glucose monitoring

#### 2.3.1 Accuracy and precision of glucose metres

Accuracy refers to the degree of consistency between the results by glucose metres and from laboratory measurements. Precision refers to the degree of agreement after repeated measurements of the same sample. In April 2021, the National Health Commission of the People's Republic of China issued the healthcare industry standard: *Guidelines for Clinical Operation and Quality Management of Portable Glucose Meters* (WS/T781‐2021).^4^ This standard has followed the International Organization for Standardization (ISO) 15197 (2013) standard for the accuracy and precision of glucose metres.^5^

1. Requirements for accuracy: 95% test results within ±0.83 mmol/L for blood glucose (BG) < 5.5 mmol/L; 95% test results within ±15% for BG ≥ 5.5 mmol/L.^4^
2. Requirements for precision: the standard deviation of the results should be <0.42 mmol/L for BG < 5.5 mmol/L; the coefficient of variation (CV) of the results should be <7.5% for BG ≥ 5.5 mmol/L.^4^

#### 2.3.2 Factors affecting testing results

Glucose oxidase monitors are sensitive to the oxygen available. Glucose dehydrogenase–based monitors are susceptible to other carbohydrates such as xylose, maltose, galactose etc.

The patient's haematocrit level has a great influence on the test results. As the haematocrit level increases, the result using the whole blood sample decreases gradually at a given plasma glucose level. The blood glucose metre with haematocrit correction can minimise this influence.

Other common endogenous and exogenous factors that may interfere with glucose readings include vitamin C, salicylic acid, uric acid, bilirubin, and triglycerides. In addition, to keep the glucose metres and the test strips in the best working condition, there are certain requirements for the temperature, humidity, and altitude of the environment.

#### 2.3.3 Differences between capillary and venous glucose levels

Usually, capillary whole blood is used for measurements by glucose metres, while venous plasma or serum samples are used for laboratory measurements. A plasma‐calibrated metre will report a reading close to the laboratory result at fasting status, but a slightly higher reading than the venous glucose result at postprandial status or after glucose load.^6^ A whole‐blood calibrated metre will report a 12% or around lower reading than that measured in the laboratory at fasting status, but a reading close to venous plasma glucose levels at postprandial status or after glucose load.^7^

### 2.4 Principles of capillary glucose monitoring

The pattern and frequency of capillary glucose monitoring should be individualised based on patients' glycaemic levels, treatment plans, and practical needs.^8^

Glucose monitoring can be applied at different time points of the day, including premeal, 2‐h postmeal, at bedtime, and night (usually 2:00 AM to 3:00 AM early in the morning). The indications for glucose monitoring at various time points are listed in Table 2.

**TABLE 2:** The optimal indications of glucose monitoring in different time points

Time points	Indications
Premeal	Patients with high fasting glucose levels, or patients with increased risk of hypoglycaemia (elder patients, patients with strict glycaemic control)
2‐h postprandial	Patients with controlled fasting glucose levels but uncontrolled HbA1c level; patients aiming to understand the effects of diet and exercise on glucose levels
At bedtime	Patients on insulin therapy, especially before dinner
At night	Patients with high fasting glucose levels but controlled glucose levels at other time points after treatment; or those with possible nocturnal hypoglycaemia
Anytime	Patients with symptoms of hypoglycaemia; prior to and after strenuous exercise

Abbreviation: HbA1c, glycated haemoglobin A1c.

Patients treated with oral hypoglycaemic drugs can monitor their glucose levels 2–4 times a week including fasting glucose or postprandial glucose levels. Patients on insulin therapy should monitor their glucose levels more actively. Patients with basal insulin therapy should pay more attention to fasting glucose levels, and patients with premixed insulin therapy should focus on fasting and predinner glucose levels. When hypoglycaemia is suspected, capillary blood glucose should be tested immediately. When the difference between the capillary and the venous blood glucose increases, attention should be paid. In addition, glucose monitoring should be added before exercise or performing critical tasks (such as driving) as needed. For patients under special medical conditions, such as perioperative patients, patients at high risk of hypoglycaemia, critically ill patients, elderly patients, patients with type 1 diabetes, and gestational diabetes, individualised glucose monitoring patterns should be implemented.

### 2.5 Patient education

Patient education includes standardised testing, recording, and interpretation of the glucose results. Currently, the proportion of patients with diabetes who perform glucose monitoring and the frequency of glucose monitoring in China are not ideal yet.^9^ It is necessary to educate people with diabetes about the importance of glucose monitoring. Medical providers should discuss and help analyse the testing results with patients and make corresponding adjustments of lifestyle and treatment plans. By doing so, glucose monitoring can become an effective self‐management tool.

The glucose log should contain various information, such as glucose levels, diet, exercise etc. If possible, use relevant software for the management of glucose data to comprehensively evaluate the trends of glycaemic control, the influence of medications, diet, and exercise on glucose levels, and to guide the adjustment of treatment. With the application and popularisation of terminal equipment such as mobile phones and computers, there are increasing scenarios for the application of information technology in medical care, and network‐based mobile health is showing a rapid development trend. In terms of diabetes management, the mobile health system can record the patients' information on glucose monitoring easily. A previous study shows that the use of the above methods can improve the modification of patients' lifestyle and patients' glucose control and provide individualised diabetes management.^10^

### 2.6 Non‐invasive glucose monitoring and its perspective

In recent years, novel technologies such as near‐infrared, mid‐infrared, Raman and other spectroscopy technologies, transdermal dialysis, metabolic heat conformation, and the multi‐parameter algorithm have been developed for noninvasive glucose monitoring through finger‐clamping, earlobe‐clamping, and other testing methods. However, only a few devices have been approved for marketing. The accuracy of the noninvasive glucose monitoring system and the lag time regarding glucose values between the novel and conventional methods are the biggest challenges for their applications in clinical practice.^11^

### 2.7 Capillary glucose monitoring and diabetic complications

Accurate and standardised capillary glucose monitoring can improve metabolic control and may reduce risk of diabetes‐related outcomes. Evidence has shown that intensive insulin therapy guided by frequent SMBG delays the onset and slows the progression of microvascular complications in patients with type 1 diabetes.^12^ SMBG is also associated with decreased diabetes‐related morbidity and all‐cause mortality in type 2 diabetes.^13^

### 2.8 Limitations of capillary glucose monitoring

It is not recommended to use capillary glucose monitoring under clinical conditions with microcirculatory obstruction at the blood sampling site, such as shock, severe hypotension, diabetic ketoacidosis, hyperglycaemic hyperosmolar states, severe dehydration, and oedema etc. Finger pricking may cause discomfort to patients. Wrong operations may also affect the accuracy of results. When the frequency of capillary glucose monitoring is insufficient, the estimation of mean glucose levels, glycaemic variability, or the incidence of hypoglycaemia should be interpreted with caution. On the other hand, frequent testing may cause some patients to feel anxious.

## 3 HbA1c

*Recommendations*

1. People with diabetes should measure HbA1c every 3 months before achieving HbA1c target, and every 6 months after the HbA1c goal is achieved (B).
2. HbA1c can be used as a supplementary criterion for the diagnosis of diabetes (B).

Glycohaemoglobin is formed by the nonenzymatic glycation of the N‐terminal amino acid on the *β* chain of haemoglobin. First, glucose forms a labile and readily reversible aldamine (Schiff base) with the N‐terminal valine on the *β* chain. The aldamine then undergoes an Amadori rearrangement to form a stable ketoamine. HbA1c reflects the average glucose levels over the past 2–3 months. HbA1c is a well‐established marker for assessing long‐term glycaemic control in diabetes and is widely used in clinical practice for the adjustment of glucose‐lowering treatment.^14^

### 3.1 Methods of HbA1c measurement

The measurement of HbA1c can be divided into two categories in terms of analytical techniques. One is based on the difference in charge carried by glycosylated and non‐glycosylated haemoglobin, such as ion exchange high‐performance liquid chromatography (HPLC) and capillary electrophoresis method, both commonly used in laboratory. The other is based on structural differences, such as boronate affinity HPLC, immunoassay etc.^15^ Regardless of the methods used, the component of HbA1c in glycohaemoglobin should be used for the testing result.

### 3.2 Standardisation of HbA1c measurement

The American Association for Clinical Chemistry established a subcommittee in 1993 to work on the standardising of HbA1c assays so that the results of different methods can be traced back to Diabetes Control and Complications Trial (DCCT) results. In 1996, the National Glycohemoglobin Standardization Program (NGSP) in the USA completed the standardisation work. In 1995, the International Federation of Clinical Chemistry and Laboratory Medicine (IFCC) established a dedicated working group to study a traceable HbA1c reference system.^16^ According to the international consensus issued in 2007 and 2010,^17^, ^18^ the reference system of IFCC is the only recognised reference system for standardised HbA1c measurement. The international unit of HbA1c is ‘mmol/mol’ and the common unit is ‘%’ of the NGSP system, both of which can be converted to each other.

Although the standardisation of HbA1c in China started late, it is making progress rapidly. In 2015, the National Health and Family Planning Commission of the People's Republic of China (now called the National Health Commission of the People's Republic of China) issued the health industry standard: *Measurement of Hemoglobin A1c*.^19^ The number of laboratories participating in the National External Quality Assessment program organised by the National Center for Clinical Laboratories increased from 313 in 2008 to 3193 in 2021, and the average inter‐lab coefficient of variation decreased from 7.3% to 3.1%. Currently, there are three IFCC network laboratories.^20^ These laboratories contribute greatly to the work of standardisation of HbA1c testing and the application of HbA1c in the diagnosis and clinical management of diabetes in China.

We recommend in the guideline that:

1. Laboratories should be equipped with IFCC and/or NGSP‐certified instruments and the relevant kit.
2. Quality control in laboratories should be strictly performed, and staff should actively participate in external quality assessment programs and glycohaemoglobin standardisation programs organised by the health administrative departments.

### 3.3 Clinical applications of HbA1c

#### 3.3.1 Evaluation of glycaemic control in people with diabetes

According to the *Guideline for the prevention and treatment of type 2 diabetes mellitus in China (2020 edition)*,^8^ HbA1c testing should be performed at least once every 3 months at the beginning of treatment and once the target is met, it can be done every 6 months. HbA1c measurements should be based on results that can be traced back to the values in DCCT. The *Guideline for the prevention and treatment of type 2 diabetes mellitus in China (2020 edition)* listed the glycaemic target of HbA1c for patients with type 2 diabetes.^8^

#### 3.3.2 Diagnosis of diabetes

In order to be in line with the standards of WHO,^21^ together with the greatly improved standardisation of HbA1c measurement across China during recent years, the *Guideline for the prevention and treatment of type 2 diabetes mellitus in China (2020 edition)* ^8^ recommends that HbA1c ≥ 6.5% can be used as a supplementary diagnostic standard in medical institutions with standardised measurement and strict quality control. However, under specific conditions, such as sickle cell disease, pregnancy, glucose‐6‐phosphate dehydrogenase deficiency, acquired immune deficiency syndrome, haemodialysis, recent blood loss or blood transfusion, as well as erythropoietin therapy, only intravenous plasma glucose levels can be used to diagnose diabetes. Also, it is not recommended to use HbA1c for the screening and diagnosis of cystic fibrosis‐related diabetes.^22^

#### 3.3.3 HbA1c and diabetic complications

According to the United Kingdom Prospective Diabetes Study and DCCT studies, HbA1c is strongly associated with clinical outcomes in patients with type 1 and type 2 diabetes. The reduction in HbA1c can effectively reduce the risk of diabetic complications such as retinopathy, nephropathy, and neuropathy.^23^, ^24^ In addition, even in populations with impaired glucose regulation, HbA1c can reflect the risk of subclinical atherosclerosis.^25^

### 3.4 Advantages of HbA1c

HbA1c is a very stable chemical with little variability. There is compelling evidence that HbA1c is closely related to the risk of chronic complications of diabetes.^14^, ^26^, ^27^ HbA1c testing reflects long‐term glycaemic control, and the result is not affected by short‐term diet, exercise, and other lifestyle modifications. It is also more convenient for patients to take blood samples at any time as the test does not require fasting status.

### 3.5 Factors affecting HbA1c testing

The factors that affect HbA1c testing can be summed up into two categories. One refers to those interfering factors unrelated to the analytical method, which can change the production and lifespan of erythrocytes, the glycosylation of haemoglobin, and the structure of haemoglobin. Any factors causing decreased erythrocyte production and prolonged lifespan of erythrocytes (e.g., iron and vitamin B12 deficiency, spleen removal etc.) will increase the HbA1c result. Conversely, increased numbers of erythrocytes in the circulation and decreased lifespan of erythrocytes (e.g., the administration of erythropoietin, iron agents, vitamin B12, chronic liver disease, spleen enlargement etc.) will reduce HbA1c.^28^ In addition, due to increased red blood cell turnover during pregnancy, the HbA1c level is slightly lower in women with pregnancy than in normal nonpregnant women.^29^ The other category refers to factors affecting the specificity of the method or assay‐related artifacts.^30^ These factors include but are not limited to high haemoglobin F level, carbamylated haemoglobin, hypertriglyceridemia, hyperbilirubinaemia, as well as haemoglobin disease or haemoglobin C, D, E, S.^31^ In addition, the administration of some medications such as high doses of vitamin C and E, high doses of salicylic acid, erythropoietin, and anti‐retroviral drugs may lower the result of HbA1c testing.^28^, ^30^

### 3.6 Limitations of HbA1c

HbA1c is limited in reflecting hypoglycaemia and glycaemic variability. Also, it has a ‘delayed effect’ when evaluating the efficacy of treatment after recent adjustment.

## 4 GA

*Recommendations*

GA is superior to HbA1c in capturing short‐term glucose changes, which is useful for evaluating the patient's short‐term glycaemic control (B).

Glycated serum protein (GSP) is the product of a nonenzymatic reaction between glucose and serum protein (albumin representing about 70% of the total proteins) in the blood. GSP levels reflect the average glucose levels over 2–3 weeks prior to testing. GSP testing is simple, fast, and does not require special equipment, which can be widely used in the primary care setting. GA is calculated as the percentage of glycated albumin to total albumin, and the serum albumin levels has no influence on the final test results and therefore is more accurate than GSP.^32^

### 4.1 Measurement of GA

Currently, a method of enzymatic detection with liquid reagents is widely used for GA measurement. This method features with good repeatability, regent stability, and relatively low cost. The glycosylated albumin and albumin content can be detected at the same time. The results were the calculation of percentage of glycated albumin in total albumin.^33^

### 4.2 Standardisation of GA measurement

Participating in the relevant external quality assessment programs can be an effective way to improve the quality of testing. From the HPLC method in the 1980s to the enzymatic method in recent years, the measurement of GA has become simpler and faster, with improved accuracy and usefulness. However, there are no well‐established international standards for its measurement and no standardised reference substances at this moment.

### 4.3 The reference value of GA

Currently, there is no recognised normal reference value of GA. In 2009, researchers from Shanghai Jiao Tong University Affiliated Sixth People's Hospital carried out a multicentre study including 10 centres across China. A total of 380 people with normal glucose regulation aged 20–69 were enrolled and the reference value of GA in the Chinese population was established as 10.8%∼17.1%.^34^ A study conducted in Beijing, China, during the same period showed that the normal reference value of GA was 11.89%∼16.87%.^35^

### 4.4 Clinical applications of GA

#### 4.4.1 Evaluation of short‐term glycaemic control

Because albumin has a short half‐life in the body and the binding speed of albumin to glucose is faster than haemoglobin, GA is more sensitive to short‐term changes in glucose levels than HbA1c and is a good indicator for short‐term glycaemic control. For patients with diabetes after recent adjustment of the treatment plan, GA was shown to have greater clinical value than HbA1c.^36^

#### 4.4.2 Identification of stress‐induced hyperglycaemia

When an acute illness such as trauma, infection, and acute cardiovascular and cerebrovascular events occurs, it is difficult to distinguish the stress‐induced hyperglycaemia from preexisting diabetes in a patient without evidence of prior diabetes. A study from China suggested that the cutoff point of using GA to differentiate between latent diabetes and stress‐induced hyperglycaemia is 17.5%.^37^ Combined assessment of GA and HbA1c can be used to determine the duration and severity of hyperglycaemia.

#### 4.4.3 Screening for diabetes

A study by Shanghai Jiao Tong University Affiliated Sixth People's Hospital suggested that GA was also suitable for diabetes screening. Most undiagnosed patients with diabetes can be detected when they present with a GA of ≥17.1% and the combined assessment of fasting plasma glucose and GA improves the detection of diabetes in Chinese subjects.^38^ An elevated GA level is an important indicator suggesting people who are at a high risk of diabetes to undergo an oral glucose tolerance test, especially for those with normal fasting blood glucose.

#### 4.4.4 GA and diabetic complications

Evidence has shown that GA, as an important glycosylation product, has a significant correlation with chronic diabetes‐related complications such as nephropathy, retinopathy, and atherosclerosis.^39^

### 4.5 Advantages of GA

Since serum albumin has multiple glucose binding sites, the production of GA is fast and efficient. Meanwhile, serum albumin has a faster turnover (short half‐life) than HbA1c, making GA a more sensitive marker to the glycaemic changes. In patients with certain diseases affecting the lifespan of red blood cells (e.g., end‐stage renal disease on haemodialysis), the value of HbA1c will underestimate the actual glucose levels of the patient, while the GA measurement is not affected, indicating that GA can better reflect glycaemic control than HbA1c in these patients.^40^

### 4.6 Factors affecting GA testing

The turnover speed of albumin affects the level of GA values. For the same glucose levels, individuals with accelerated albumin turnover have lower GA levels, and vice versa. Therefore, these factors should be taken into consideration when interpreting the GA levels of patients with diseases associated with abnormal albumin homoeostasis (such as nephrotic syndrome, abnormal thyroid function and liver cirrhosis).

Body mass index is inversely associated with GA.^41^ The effect of increased body fat on GA levels may be through some mechanisms related to fat mass and visceral adipose tissue.^42^ Possible explanations include increased turnover and catabolic rate of albumin and chronic low‐grade systemic inflammation in obese subjects. In the clinical use of GA, it should be noted that in people with increased body fat or central obesity, GA may underestimate the actual blood glucose level.

Thyroid hormones can promote the decomposition of albumin and affect the level of serum GA. Hyperthyroidism may lower the values of GA, while hypothyroidism may increase the values. Even in people with normal thyroid function, GA is negatively correlated with serum free triiodothyronine and free thyroxine.^43^

### 4.7 Limitations of GA

Similar to HbA1c, GA reflects average glucose levels and does not provide actionable information on hypoglycaemia and glycaemic fluctuations. GA levels should be interpreted cautiously in disorders with abnormal albumin turnover, such as nephrotic syndrome, liver cirrhosis etc.

## 5 1,5‐ANHYDROGLUCITOL

*Recommendations*

Serum 1,5‐anhydroglucitol can be used as an auxiliary marker of glucose monitoring (B).

1,5‐Anhydroglucitol (1,5‐AG), the C‐1 deoxy form of glucofuranose, reflects the average glucose level over the previous 1–2 weeks. It is significantly decreased in patients with diabetes and is superior to other glucose indicators in monitoring postprandial hyperglycaemia.^44^ In 2003, the U.S. Food and Drug Administration approved the launch of a kit for testing serum 1,5‐AG.^45^, ^46^ Serum 1,5‐AG can be used as an auxiliary glucose monitoring metric to guide the adjustment of treatment plans.^47^, ^48^ In recent years, researchers from China have established an accurate mass spectrometry method for saliva 1,5‐AG, which provides new insights into the noninvasive monitoring and screening of diabetes in the future.^49^ The current evidence linking 1,5‐AG to diabetic complications remains scarce. In a cross‐sectional analysis of 1600 participants from the Atherosclerosis Risk in Communities Study, serum 1,5‐AG was significantly associated with retinopathy and albuminuria.^50^

## 6 CGM

*Recommendations*

1. CGM can detect hyperglycaemia and hypoglycaemia that are not easily recognised by traditional monitoring methods. (A)
2. Time in range (TIR) is a useful metric to assess glycaemic control in people with diabetes. (B)

CGM refers to the technology that continuously measures glucose concentrations in the subcutaneous interstitial fluid by glucose sensors. It provides comprehensive information on glucose values through day and night, thereby facilitating the identification of the trends and characteristics of glucose profiles. Therefore, CGM is promising to be an effective supplement to traditional blood glucose monitoring methods. With the advancement of the technology and the advent of new devices in recent years, CGM has been increasingly recognised and accepted by clinicians and patients, which shows a good prospect of clinical applications.

### 6.1 Clinical characteristics of CGM

#### 6.1.1 Types of CGM devices

According to the characteristics of different systems, CGM devices currently available can be divided into retrospective CGM, real‐time CGM, and flash glucose monitoring (FGM).

1. **Retrospective CGM**: Retrospective CGM cannot display the glucose levels of the user in real time. Relevant data must be downloaded after monitoring, so it is also called blinded CGM. Retrospective CGM is valuable for identifying trends and patterns of glucose profiles, which can guide behaviour changes and/or treatment adjustments and is particularly useful in patients with type 1 diabetes, type 2 diabetes on intensive insulin therapy, and patients with increased glucose fluctuations. In addition, as the glucose data is blinded during monitoring, this system can avoid excessive interventions from the users and/or clinicians and reflect the glucose status of users in daily life objectively. Therefore, retrospective CGM is an important tool for CGM‐related clinical studies. Using retrospective CGM, a national multicentre study in China established the normal reference range for CGM and recommended that the 24 h mean blood glucose value <6.6 mmol/L, mean amplitude of glucose excursions <3.9 mmol/L, and standard deviation (SD) of blood glucose <1.4 mmol/L be the normal CGM reference range for the Chinese population.^51^, ^52^
2. **Real‐time CGM**: Compared with retrospective CGM, the main features of real‐time CGM include: (1) providing real‐time glucose values; (2) alarms and alerts for high or low glucose values; and (3) displaying the trend of glucose change (represented by trend arrows). Therefore, real‐time CGM is suitable for patients with large glucose fluctuations and a high risk of hypoglycaemia, especially for those with recurrent nocturnal hypoglycaemia and/or asymptomatic hypoglycaemia. In patients with type 1 diabetes treated with multiple daily insulin injections or continuous subcutaneous insulin infusion, there is convincing evidence that the use of real‐time CGM can significantly reduce HbA1c and the risk of hypoglycaemia.^53^, ^54^ Data also exists to support the beneficial effect of real‐time CGM on glucose control in type 2 diabetes.^55^
3. **Flash glucose monitoring (FGM)**: FGM belongs to the category of intermittently scanned CGM and was the only system available of its kind at the time of this writing. Unlike the real‐time CGM, which continuously and automatically provides real‐time glucose values, the FGM displays glucose values only when swiped by a reader or a smartphone. The system does not require SMBG calibration, therefore avoiding the pain of frequent finger prick and may help improve the patient's compliance with glucose monitoring. Several randomised controlled clinical trials have shown that FGM may improve glucose control in patients with type 1 diabetes and those with type 2 diabetes on insulin therapy.^56^, ^57^

#### 6.1.2 Main advantages of CGM and indications for CGM

The main advantage of CGM lies in its ability to detect hyperglycaemia and hypoglycaemia that could barely be identified by traditional monitoring methods, especially postprandial hyperglycaemia and nocturnal asymptomatic hypoglycaemia.^58^ Examples include: (1) recognising glucose fluctuations caused by factors such as food, exercise, treatment, and psychological status; (2) identifying postprandial hyperglycaemia, nocturnal hypoglycaemia, and dawn phenomenon and Somogyi phenomenon that are difficult to be detected by traditional blood glucose monitoring methods; (3) informing on individualised treatment plans; (4) improving treatment compliance; and (5) providing a means of visualised diabetes education.

Based on the advantages of CGM and existing clinical evidence, real‐time CGM or FGM is recommended in patients with type 1 or type 2 diabetes treated with intensive insulin therapy who are willing and able to use the device. Additionally, CGM should be considered in patients with diabetes experiencing problematic hypoglycaemia (i.e., frequent hypoglycaemia, nocturnal hypoglycaemia, or hypoglycaemia unawareness). As a relatively new monitoring technique, CGM has also been used in specific groups of individuals in the Chinese population such as prediabetes, fulminant type 1 diabetes, monitoring of nocturnal hypoglycaemia in elderly patients with diabetes and concomitant cardiovascular and cerebrovascular diseases, and in patients with diabetes complicated by infection.^59^, ^60^, ^61^, ^62^, ^63^, ^64^, ^65^

Each CGM system has unique features including sensor lifespan, frequency of readings, warm‐up time, mean absolute relative difference (MARD), need for calibration, alarms/alerts etc. Among these parameters, MARD is used for assessing sensor accuracy. It is generally accepted that a MARD of <10% is sufficiently accurate to make diabetes treatment decisions (i.e., ‘non‐adjunct’ use of CGM as a replacement for SMBG). However, none of the available CGM systems has been approved for the non‐adjunct use in the Chinese market. Importantly, at least 14 days of active CGM usage are needed for the optimal evaluation of glucose status,^66^ and the patient should calibrate the sensor, if needed, according to manufacturer instructions to ensure accuracy. Of note, CGM is still relatively expensive. Although CGM was previously reported to be cost‐effective compared to SMBG in patients with type 1 or type 2 diabetes on insulin therapy,^67^, ^68^, ^69^, ^70^ these studies were mostly conducted in developed countries, and relevant data in China is scarce. Therefore, it is important that the initiation of CGM should be based on the thorough evaluation of a patient's characteristics, needs, and availability of devices and shared decision‐making.

### 6.2 Clinical applications of CGM

#### 6.2.1 Patient education and training

The willingness and ability of patients to use the CGM system are directly related to the efficacy of CGM. Therefore, it is recommended to provide adequate and effective education and training for patients before the use of CGM (especially in the ambulatory setting) and continued support to problems that may arise during the long‐term use. This helps increase CGM adherence, thereby improving glucose control. It is worth mentioning that SMBG still plays an important role during CGM use. Besides being used for the calibration of some CGM systems, capillary blood glucose testing should be performed to make treatment decisions when CGM suggests hypoglycaemia, when a patient suspects hypoglycaemia, or when there is discordance between symptoms and sensor readings.

#### 6.2.2 The interpretation of the CGM report

The international TIR consensus recommended the ambulatory Glucose Profile (AGP) as the standardised reporting form for CGM data.^66^ AGP aggregates glucose data over multiple days into a 24‐h mode. The overall glucose profile is visualised by smoothed curves representing the frequency percentiles (5th, 25th, 50th, 75th, and 95th percentiles) of glucose values, which indicates the inter‐day variability. When interpreting AGP results, it is important to look for glucose patterns and trends, instead of the absolute glucose readings at certain time points, and try to find potential causes for abnormal glucose fluctuations. It is suggested that the first step is to identify hypoglycaemia, the next step is to seek for hyperglycaemia, followed by the analysis of glycaemic variability (including intra‐day and inter‐day glycaemic variability). Finally, treatment adjustments should be made after discussing with the patient to address the problems in the glucose profile.^71^

#### 6.2.3 Key metrics of the standardised CGM report

The wealth of data generated by the CGM system provides great convenience for comprehensively evaluating the quality of glucose control in diabetes. The *International Consensus on Use of CGM* recommended 14 parameters as the core CGM metrics in standardised reports.^72^ Of them, 10 metrics including TIR, Time Above Range, and Time Below Range (TBR) have great value for clinical evaluation of glucose control.^66^ TIR refers to the time (expressed in minutes) or the percentage (expressed in %) of glucose within the target range (usually 3.9–10.0 mmol/L in non‐gestational adults) within 24 h. Currently, the recommended target for TIR control in most patients with type 1 diabetes and type 2 diabetes is >70%, with a TBR (<3.9 mmol/L) of <4% and a TBR (<3.0 mmol/L) of <1%, and the individualisation of glycaemic targets should be emphasised.^66^ The CV of glucose levels, defined as (SD/mean glucose) × 100%, is recommended as the core parameter for assessing glycaemic variability.^72^ The main advantage of CV lies in its simplicity and its independence of mean glucose. There is evidence that a CV of >33% was related to excess risk of hypoglycaemia in a Chinese population with diabetes.^73^ Mean glucose is an important measure reflecting the overall exposure to hyperglycaemia, which can be converted to an estimated HbA1c using a population‐based formula. To reduce the confusion for clinicians and patients when the estimated HbA1c does not match with the laboratory‐measured HbA1c, the term ‘glucose management indicator’ (GMI) is now used to replace ‘estimated HbA1c’. From a clinical perspective, the potential discordance between GMI and HbA1c could help inform diabetes management.^74^ For instance, a greater difference between HbA1c and GMI was associated with higher risk of hypoglycaemia in a non‐White population.^75^

### 6.3 CGM metrics and diabetes‐related outcomes

Previous observational studies reported that TIR was significantly associated with microvascular complications,^76^, ^77^, ^78^ a surrogate marker of cardiovascular disease,^79^ neonatal outcomes,^80^ and all‐cause and cardiovascular mortality,^81^ suggesting that TIR can be a useful indicator for assessing glucose control. In a recent prospective study conducted in Chinese patients with seemingly well‐controlled type 2 diabetes, CV was reported to be significantly linked to all‐cause mortality.^82^

### 6.4 Limitations of CGM

Since CGM measures glucose levels in the interstitial fluid instead of blood, there is a time lag between CGM readings and blood glucose values,^83^ which is more evident when blood glucose changes rapidly. In addition, the accuracy of CGM tends to deteriorate in the hypoglycaemic range.^84^, ^85^ The typical wear‐time of CGM sensors on the market ranges from 3 to 14 days. Therefore, glucose metrics generated by CGM only reflects the short‐term glucose control. Furthermore, there is a lack of standardisation across CGM systems, and the data on head‐to‐head comparisons of CGM sensors are limited,^86^, ^87^, ^88^ making it impossible to directly compare the CGM parameters reported by different studies. Finally, the relatively high cost of CGM poses a major obstacle to the utilisation of CGM in clinical practice.

## 7 CONCLUSIONS

In conclusion, blood glucose monitoring is an integral part of diabetes management. Different metrics of glucose monitoring have their own strengths and reflect different aspects of glucose control, which cannot be replaced by one another (Table 3). Given the available evidence, recommendations for the use of common monitoring methods in clinical practice are summarised in Table 4. These monitoring methods should be selected and combined according to the characteristics of patients for the optimal evaluation of glucose status, thereby guiding the clinical decision‐making.

**TABLE 3:** Characteristics and clinical applications of common glucose monitoring methods

Methods	Clinical significance	Clinical applications	Potential drawbacks
Capillary blood glucose	Reflects glucose level at a time point	Basic form of blood glucose monitoring. The timing and frequency can be individualised according to the patient's condition and treatment plan.	Accuracy is dependent on both the glucose metre and user. Finger pricking may cause discomfort.
HbA1c	Reflects glucose level over the previous 2–3 months	Important for the adjustment of treatment and the assessment of risk of diabetic complications. HbA1c ≥ 6.5% is a supplementary criterion for the diagnosis of diabetes.	Some conditions may affect testing results. Does not provide information on hypoglycaemia and glycaemic fluctuations.
GA	Reflects glucose level over the previous 2–3 weeks	Assessment of short‐term glucose control. It may be helpful for the identification of stress‐induced hyperglycaemia.	Limited evidence. Some conditions may affect testing results. Does not provide information on hypoglycaemia and glycaemic fluctuations.
CGM	Reflects continuous and comprehensive blood glucose data	Presenting the trends and patterns of glucose profiles. It could be used to detect hyperglycaemia and hypoglycaemia that are not recognised by traditional monitoring methods, especially postprandial hyperglycaemia and asymptomatic nocturnal hypoglycaemia.	Limited but increasing evidence. Limited accessibility in some areas. Relatively high cost. Difficulties in interpreting the report.

Abbreviations: CGM, continuous glucose monitoring; GA, glycated albumin; HbA1c, glycated haemoglobin A1c.

**TABLE 4:** Recommendations for the use of common methods of glucose monitoring in clinical practice

Capillary blood glucose	Self‐monitoring of blood glucose (SMBG) is an integral part of diabetes management and education. It is recommended that all patients with diabetes should perform SMBG (A).
	Individualised protocols of capillary glucose monitoring should be made according to the practical needs of patients with diabetes (B).
HbA1c	People with diabetes should measure HbA1c every 3 months before achieving HbA1c target, and every 6 months after the HbA1c goal is achieved (B).
	HbA1c can be used as a supplementary criterion for the diagnosis of diabetes (B).
GA	GA is superior to HbA1c in capturing short‐term glucose changes, which is useful for evaluating the patient's short‐term glycaemic control (B).
1,5‐AG	Serum 1,5‐anhydroglucitol can be used as an auxiliary marker of glucose monitoring. (B)
CGM	CGM can detect hyperglycaemia and hypoglycaemia that are not easily recognised by traditional monitoring methods. (A)
	Time in range (TIR) is a useful metric to assess glycaemic control in people with diabetes. (B)

Abbreviations: 1,5‐AG, 1,5‐anhydroglucitol; CGM, continuous glucose monitoring; GA, glycated albumin; HbA1c, glycated haemoglobin A1c.

## AUTHOR CONTRIBUTIONS

Yuqian Bao and Dalong Zhu designed the guideline's scope, made the schedule, and supervised the whole project. All members of guideline writing group participated in the writing, discussion, and revision of this paper. All members of expert committee critically reviewed the article. All members of guideline writing group and expert committee have read and approved the final manuscript.

## CONFLICT OF INTEREST

The authors declare that they have no conflict of interest.

## ETHICS STATEMENT

The study did not involve human or animal subjects and therefore no approval was required.

### PEER REVIEW

The peer review history for this article is available at https://publons.com/publon/10.1002/dmrr.3581

## MEMBERS OF THE GUIDELINE WRITING GROUP

Yuqian Bao (Shanghai Jiao Tong University Affiliated Sixth People's Hospital), Jian Zhou (Shanghai Jiao Tong University Affiliated Sixth People's Hospital), Yufei Wang (Shanghai Jiao Tong University Affiliated Sixth People's Hospital), Yingsheng Zhou (Beijing Anzhen Hospital, Capital Medical University), Hua Bian (Zhongshan Hospital, Fudan University), Qi Pan (Beijing Hospital), Zhifeng Cheng (The Fourth Affiliated Hospital of Harbin Medical University), Jingyi Lu (Shanghai Jiao Tong University Affiliated Sixth People's Hospital), Yifei Mo (Shanghai Jiao Tong University Affiliated Sixth People's Hospital).

## MEMBERS OF EXPERT COMMITTEE

Weiping Jia (Shanghai Jiao Tong University Affiliated Sixth People's Hospital), Dalong Zhu (Drum Tower Hospital Affiliated to Nanjing University Medical School), Lixin Guo (Beijing Hospital), Liming Chen (Tianjin Medical University Chu Hisen‐I Memorial Hospital), Li Chen (Qilu Hospital of Shandong University), Qiuhe Ji (Xijing Hospital, Air Force Military Medical University), Xiaoying Li (Zhongshan Hospital, Fudan University), Xingwu Ran (West China Hospital of Sichuan University), Zhiming Zhu (Daping Hospital, Army Medical University), Yaoming Xue (Nanfang Hospital, Southern Medical University), Minxiang Lei (Xiangya Hospital Central South University), Guangda Xiang (General Hospital of Central Theater Command of People's Liberation Army), XiangJin Xu (900 Hospital of the Joint Logistics Team), Jianhua Ma (Nanjing First Hospital, Nanjing Medical University), Yiming Li (Huashan Hospital, Fudan University), Yuqian Bao (Shanghai Jiao Tong University Affiliated Sixth People's Hospital), Hongyu Kuang (The First Affiliated Hospital of Harbin Medical University), Ling Li (Shengjing Hospital of China Medical University), Jing Yang (First Hospital of Shanxi Medical University), Guangyao Song (Hebei General Hospital), Xueyao Han (Peking University People's Hospital), Jingtao Dou (The First Medical Center, Chinese PLA General Hospital), Xinhua Xiao (Peking Union Medical College Hospital), Qiu Zhang (The First Affiliated Hospital of Anhui Medical University), Qing Su (Xinhua Hospital Affiliated to Shanghai Jiao Tong University School of Medicine), Lixin Shi (The Affiliated Hospital of Guizhou Medical University), Jing Liu (Gansu Provincial Hospital).

## Data Availability

Data sharing is not applicable to this article as no new data were created or analysed in this study.
